# A novel wide scale well-baby clinic mobile application: an Egyptian pilot study

**DOI:** 10.1186/s12913-023-09720-0

**Published:** 2023-06-24

**Authors:** Noha M. Ibrahim, Hanan S. Ez-Elarab, Mohamed Momen, Isis M. Mossad, Sherif S. Eletriby

**Affiliations:** 1 Department of Community, Environmental and Occupational Medicine, Faculty of Medicine, Ain Shams University, 38 Ramses St., Abbassia Square, Cairo, 11566 Egypt; 2 Faculty of Computers and Information, Menoufia University, Shebin Al Kom, Al Minufiyah Egypt

**Keywords:** mHealth, Mobile app, Well-baby clinics, Child healthcare

## Abstract

**Background:**

Utilization of under 5-year-old child healthcare services in Egypt is considered low, the highest proportion of well-baby visits is mainly for immunization in the first 2 years of age. Mobile health (mHealth) interventions have the potential to be a useful and low-cost way to disseminate information about proper nutrition, can be used to monitor children’s growth using the official charts of World Health Organization, can also help in accessing vaccine-related information and schedules.

**Objectives:**

To assess needs and requirements for a new comprehensive well-baby clinic mobile application (app) covering well-baby clinic service components. Thereafter, to develop the app prototype and validate it.

**Methods:**

This study was conducted in four phases: User requirements, development, validation and usage. In user requirement phase, the need for the new app was assessed by performing literature review, market app research and an online survey. In development phase, we developed the novel well-baby clinic app that constituted all well-baby clinic services for children’s health monitoring relying on evidence-based information and honoring data safety. In validation phase, after a series of testing, the app was validated using Mobile app rating scale (MARS) by public health and pediatrics consultants to assess its quality. Finally, the app was launched and made available to the public on Android platform.

**Results:**

*Sehhat Tefly* app was developed based on the demands and requirements of mothers of under 5-year-old children. The app constituted caregiver, child information and seven service elements: physical growth, developmental milestones, immunizations, nutrition, teething, safety & emergency measures and report. The app quality mean was rated 3.7 out of 5 by the panel of experts. The app was downloaded 1445 times in a 4 month period.

**Conclusions:**

*Sehhat Tefly* app can meet the need for a free, easy and accessible tool for caregivers to track the progress of children’s development and wellbeing. It can also provide advice for referral to physician consultation in case of deviation from normal measures.

**Supplementary Information:**

The online version contains supplementary material available at 10.1186/s12913-023-09720-0.

## Introduction

Routine checks on apparently healthy children are an important component of the preventive services available for children. Well-baby clinics located in primary health care centers (PHCs) in Egypt dated since 1926 [1]. The utilization of these services shows constant low rates. The highest proportion of well-babies (61.6%) visited the clinic only for immunization reasons [2]. The Egyptian PHC system has made significant advances in area of childhood immunizations and lesser strides in monitoring growth and child nutrition, while services for early childhood development are completely absent [3]. This creates the opportunity for mHealth to contribute to improving health care by alleviating the unequal distribution of resources as well as facilitating more efficient healthcare and delivery.

From 2020 to 2021, the number of mHealth applications (apps) available to Android users via the Google Play Store kept growing, reaching over 65,300 apps in the end 2021. In 2022, there were 54,546 healthcare and medical apps available on Google Play Store [4] compared to 41,517 apps available on the Apple App Store [5].

Mobile technologies enable the development of mHealth interventions which have the potential to be a useful and cheap way for disseminating information about proper nutrition [6]. Official charts from organizations such as the World Health Organization (WHO) [7] can be used to monitor children’s growth remotely. It can also be used to increase vaccination rates by assisting in accessing vaccine-related information, recommended immunization schedules, storing vaccination records and receiving appointment reminders [8]. The aim of this study is twofold. First, to assess needs and requirements for a new comprehensive well-baby clinic mobile app covering well-baby clinic service components. Second, to develop and validate the app to be used by caregivers of under 5-year-old children to follow their children’s health and wellbeing.

## Methods

### Study design

An interventional study conducted on caregivers of under 5-year-old children living in Egypt, targeting well-educated owners of Android smartphones who agree to participate in the study. Children who have any congenital anomalies or chronic diseases as diabetes, heart or lung diseases were excluded from the study.

### Sample size and sampling method

A sample size of 500 participants was chosen for this study as a pilot. We used a quota sample of 100 children in each age category. Recruitment of caregivers who comply with inclusion criteria and agree to participate occurred via the in-app online registration.

This study was conducted in four phases:

- **Phase 1:** Literature review, market research & user requirements.
- **Phase 2:** Application development.
- **Phase 3:** Application validation and testing.
- **Phase 4:** Application implementation and usage.

### Phase 1: literature review, market research & user requirements

Before starting the project, a preliminary research about published papers and the existing apps in the childcare field was performed. Market research (mobile app research) is the process of understanding target market, competitors, industry and current market trends. It helps in validating the app’s idea and ensures that it addresses existing market needs. It was done by searching Google play store for pre-existing Arabic apps to address their strength points, weak points, number of downloads, rating and release dates using the following keywords “baby”, “baby health”, “child health”, “child growth”, “child development”, “child teething” and “child nutrition”.

Then, user requirement assessment was done. The objective of this phase was to understand user requirements and assess customers’ needs and expectations in order to maximize the benefits and usage of the mobile app. We used an online google form sheet containing closed and open ended questions to explore participants’ frequency and location of following up their children’s growth and development. We also assessed the needs of services that should be present in the application by asking “What are the services you need most in the application?”. We also assessed previous use of children applications and asked about their feedback about them by asking “If you have ever used a mobile application for tracking children’s health, what do you think of it?”.

### Phase 2: application development

#### Mobile app designing

**Fig. 1:**
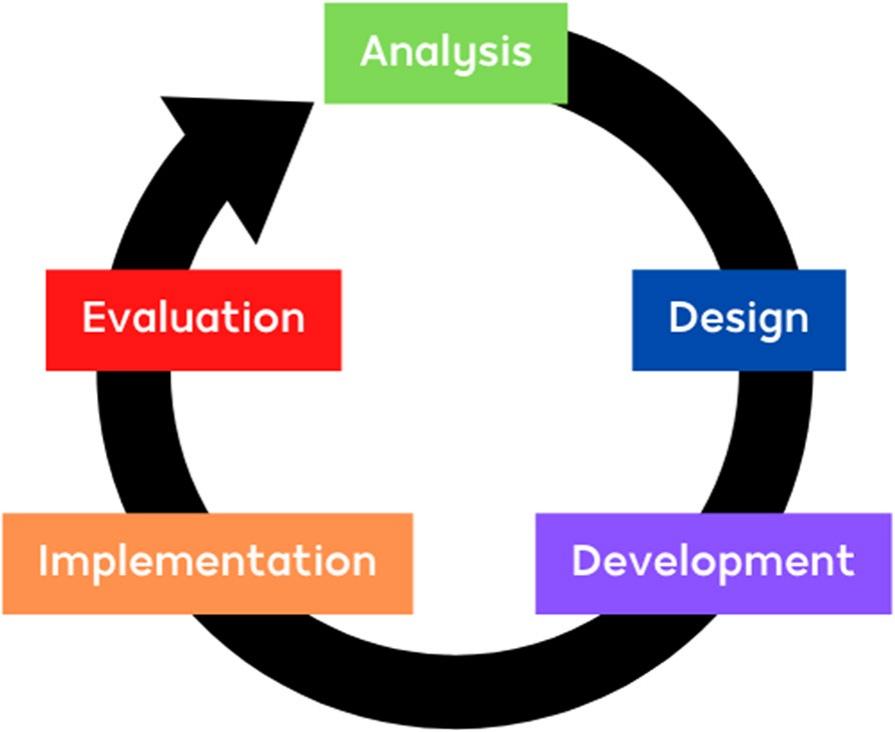
The instructional model (ADDIE) [9]

The app was designed by a team of public health, epidemiologist/statistician and computer science expert after a series of brainstorming meetings. The design was based on the general model ADDIE (Analysis Design Development Implementation Evaluation) [9] as illustrated in Fig. 1.

#### Features / screens

A user flowchart diagram was created to chart and simulate the flow of the mobile app by means of the researchers and was sent to the information technology (IT) software engineer for programming and coding. The diagram is illustrated in supplementary file 1).

#### Well-baby clinic services

The app offers seven services which were adopted from the services existed in the Egyptian PHCs and offered to under 5-year-old children [10]. These services are:

1. *Physical growth assessment*. Using basic anthropometric measurements as weight (kg), height (cm) and head circumference (cm) which are automatically plotted on WHO growth charts [7]. Indices measures as weight for age, height for age, weight for height, head circumference for age and body mass index (BMI) for age are calculated and displayed with an appropriate advice according to status of the child whether normal or abnormal.

For drawing the charts, we used the excel sheets of child growth standards on WHO website [11]. For measuring the Z scores, the reference means and standard deviations (SDs) for each child 18 visits schedule on months (2, 4, 6, 9, 12, 15, 18, 21, 24, 28, 32, 36, 40, 44, 48, 52, 56, 60) were extracted from the excel sheets of the child growth standards of the WHO [11]. The formalized excel sheet was sent to the IT (Information technology) engineer to be incorporated in the app for Z score calculation using this formula:

$$\frac{\boldsymbol{Measure}\,\left(\boldsymbol{of\,child\,data\,entered}\right)-\boldsymbol{Mean\,reference}\,\left(\boldsymbol{from\,sheets\,according\,to\,age}\right)}{\boldsymbol{SD\,reference}\,\left(\boldsymbol{from\,sheets\,according\,to\,age}\right)}$$

For height for age, children are classified according to the Z scores into above (+3) very tall and were advised to seek an endocrinologist, from (-2) to (+3) normal height for age, from (-3) to (-2) stunted and below (-3) very stunted for referral.

For weight for age, children are classified according to the Z scores into above (+1) caregiver is advised to better assess weight for height curve, from (-2) to (+1) normal weight for age, from (-3) to (-2) underweight and below (-3) very underweight for referral.

For weight for height, Children are classified according to the Z scores into above (+3) obese, from (+2) to (+3) overweight, from (+1) to (+2) at risk of overweight, from (-2) to (+1) normal weight for height, from (-3) to (-2) wasted and below (-3) severely wasted and the caregiver was advised to seek immediate medical help.

For BMI for age, Children were classified according to the Z scores into above (+3) obese, from (+2) to (+3) overweight, from (+1) to (+2) at risk of overweight, from (-2) to (+1) normal weight for height, from (-3) to (-2) wasted and below (-3) severely wasted and the caregiver was advised to seek immediate medical help. BMI was calculated using the following formula:

$$\frac{\mathit W\mathit e\mathit i\mathit g\mathit h\mathit t\mathit\;\mathit(\mathit k\mathit g\mathit)}{\mathit H\mathit e\mathit i\mathit g\mathit h\mathit t\mathit\;{\mathit(\mathit m\mathit)}\mathit\;\mathit x\mathit\;\mathit H\mathit e\mathit i\mathit g\mathit h\mathit t\mathit\;\mathit(\mathit m\mathit)}$$

For head circumference, Children are classified according to the Z scores into above (+2) macrocephaly and the caregiver was advised to seek immediate medical help, from (-2) to (+2) normal head circumference for age and below (-2) microcephaly the caregiver was advised to seek immediate medical help.

In cases of malnutrition problems, parents are asked about its causes by a number of prespecified questions. In case of obesity, overweight and at risk of overweight, user is asked about history of Cushing syndrome, growth hormone deficiency, hyperinsulinism, hypothyroidism, Down syndrome and Turner syndrome. In case of wasting, stunting and underweight, user is asked about previous history of cerebral palsy, congenital heart disease and cystic fibrosis. Thereafter, the caregiver is given advice on proper child nutrition.

2. *Developmental milestones.* Using the Arabic version of Centers of disease control and prevention (CDC) milestone development tool [12], children aging (2, 4, 6, 9, 12, 18, 24 months and 3, 4, 5 years) are assessed for: social, language, cognitive and physical development. A suitable advice and activities are given to the user according to age.
3. *Immunizations.* User can add vaccinations according to child’s age and receive an in-app reminders for the next vaccination according to the Egyptian Extended program of immunization (EPI) schedule. Also, user is asked for side effects and suitable advice is given accordingly. Side effects include a. fever where user is asked for the exact temperature and whether its rectal or axial measurement, b. redness, c. swelling, d. tenderness, e. small hard lump (nodule) at the injection site and f. sleepiness.
4. *Nutrition.* User is asked to add nutritional history of the child: a. Breastfeeding: whether its exclusive or not, regular or on demand, frequency per day. Also asked about post feeding satisfaction, problems with breasts or suckling and drugs during lactation. b. Formula milk: whether supplementary or complementary, number of feeds per day and type, concentration, amount of powder and duration of feed. c. Weaned: caregiver is asked for timing of start of weaning and what types of food introduced. Also, caregiver is asked about problems associated with weaning as anorexia, picky eating, pica, vomiting, diarrhea, constipation, respiratory or skin allergy. d. Vitamin D supplementation: caregiver is asked about number of drops given per day. Also, advice on dosing and importance of vitamin D is given. e. Iron supplementation and f. multi-vitamins supplementation.
5. *Safety & emergency measures.* Using home environmental safety questionnaire Child Injury Assessment Tool [13]. Assessment of home environmental safety regarding areas where most home injuries occur (kitchen, bathroom, living and bedroom). User is provided with health education photos on how to prevent injuries children are at risk of and how to respond in case of injuries (fractures, burns, cuts, poisoning, suffocation and drowning).
6. *Teething.* User is asked to insert date of start of teething and date of eruption of each tooth separately. The normal ranges of tooth eruption were incorporated in the mobile system according to the American dental association (ADA) [14]. Teeth care advice is illustratively given.
7. *Report.* Finally, interpretation of all provided data is illustrated with a conclusive short report.

#### Interactivity

The app contains interactive features that allowed the caregivers to record child’s data on regular basis (at each of the well-baby scheduled visits). The interpretations and advice are based on input from each individual user. Interactivity features are described in Table 1.

**Table 1:** Interactivity features in the app

**Service**	**Interactivity features**	**Instant automated feedback**
**Physical growth**	Inserting anthropometric measures	Calculation of Z score Interpretation of growth status Advice according to status
**Development**	Clicking checkbox of milestones	Red flags and advice on activities for skills development according to age
**Immunizations**	Clicking checkbox of side effects	Advice according to the side effect
**Nutrition**	Inserting nutritional history	Advice according to age
**Safety**	Clicking checkbox of any question	Advice according to the type of risk or injury
**Teething**	Inserting tooth eruption schedule	Assessment of dental eruption Advice in case of delayed teething
**Report**	New visit data	Updating the report according to data inserted in the last visit

#### Media preparation

After finalizing the structure and key aspects of the content, the researchers prepared different media for the app. Media in the app took the form of app logo, videos and images. The logo, which took the shape of a baby, was designed on Canva. The included app videos which contained health education videos were designed on Adobe premiere pro 2015. Videos included were “how to properly measure weight, height and head circumference of the child at home (Growth service)”, “how to properly clean baby’s teeth (Teething service)”, “how to measure child’s temperature to provide a valid data (Immunization service)”. Further, six “how to use the app” videos for each separate service were designed on Canva and uploaded to the app download page. As for the images used in health education messages of safety service, they were designed on Canva.

#### Database formulation

The app is linked to an online database which was formulated by the software engineer and was secured via username and password to protect users’ privacy. It consists of two parts: first part is “the dynamic app content database” or “back-end system” where data is uploaded and fed to the app by means of the researcher. The second part of the database is “Users’ information reports” which contains the list of users who registered in the app. Data are stored and easily retrieved and downloaded in the form of word file or excel file. Every child has an identification number (ID) that is fixed throughout all the services.

### Phase 3: application validation and testing

The validation phase was divided into 3 phases. The first was the Alpha testing phase which is a testing method used to unveil bugs that might arise due to unexpected errors created by the users and validate the quality state of the app’s software in minimal time. It was done by means of the research team, consisted of systematic and exhaustive procedures executing all the operations permitted by the app in several devices to assess the app performance. These devices are the following: Samsung Galaxy A52 (6.5 inches), Samsung Note 10 Lite (6.7 inches), Samsung Note 20 ultra (6.9 inches), Xiaomi Redmi Note 10 (6.4 inches) and Huawei P30 Pro (6.47 inches).

The second was Beta testing of the app which is performed by the intended audience of the app to get user feedback for the product and check if the product meets the objectives of the app. It was conducted using interviews with a non-random sample of end-users in which they emphasize their feedback and perceptions as they navigate through the services of app. These interviews were conducted to obtain preliminary user feedback, which was used to formalize the final version of the app. Participants were asked to navigate the app and were asked to use different app services and provide feedback [15].

In the third phase, the application was validated using Mobile App Rating Scale (MARS) [16] by public health and pediatrics consultants. It consists of 2 sections: app quality rating and app subjective quality. App quality rating that assesses app quality on four dimensions (engagement, functionality, aesthetics and information). App subjective quality includes the following four questions: Would you recommend this app? How many times do you think you would use this app? Would you pay for this app? What is your overall star rating of the app? MARS is scored by calculating the mean scores of the engagement, functionality, aesthetics, and information quality objective subscales, and an overall mean app quality total score.

### Phase 4: application implementation and usage

*Sehhat Tefly* (My baby’s health in English) app was developed on Android platform. The program was coded using Android Studio by the IT software engineer developer under supervision of the expert of computer science in the research team. In order to meet the specifications, set by the researchers, it was built in Java programming language, and its application programming interfaces (APIs) were supported by Google. The software programs for the Android operating system came in a format named the Android App Bundle (AAB) file. The app was developed in the Arabic language to be convenient with different sociodemographic levels of Egyptian caregivers. Of course, developing the app in English is also possible, and the researchers plan to add English language to the app in the future.

## Results

### Phase 1: market research & user requirements

Review of available mHealth revealed 13 Arabic apps. None of the apps provided any comprehensive approach to well-baby clinic services. All of them (100%∼13/13) were in the form of written articles that offered no interactive features for parents, and only offered general health education not tailored according to the status of the child. They took the form of nutritional advice, food recipes, parenting advice, static vaccination tables, weight tracking with no interpretation and sleep meter. None of them were developed by a recognized health organization. The most common service available in these apps was “nutrition service” (84.6%∼11/13) but only took the form of narrative articles or recipes. Most of them (92.3%∼12/13) included ads. They were rated from 3.7 – 4.7 stars on Google ratings. Downloads ranged from 1000+ to 500,000+ downloads. Detailed description of apps in the review is illustrated in supplementary file 2.

As for the online survey, two thirds of the mothers were in the age group of 30–39 years. Twenty-eight percent of the mothers had their youngest child range of age from 0 to less than 1 year. More than half (56.5%) of mothers monitor their children’s growth regularly, while two thirds (63.6%) of them monitor their children’s developmental milestones. Approximately sixty percent (60.7%) of mothers reported that working was one of most important factors contributing to not regularly monitoring their children’s growth (Table 2).

**Table 2:** Sociodemographic characteristics of mothers participating in needs assessment survey

	**Frequency (N.)**	**Percent (%)**
**Age of mother (in years) (*n* = 374)**		
20–29	117	31.3
30–39	249	66.7
40–50	8	2.0
**Age of youngest child (in years) (*n* = 374)**		
0–< 1	106	28.4
1–< 2	76	20.3
2–< 3	64	17.1
3–< 4	67	17.9
4–< 5	61	16.3
**Monitoring child growth (*n* = 374)**		
Yes	212	56.7
No	162	43.3
**Monitoring child development (*n* = 374)**		
Yes	238	63.6
No	136	36.4
**Location of monitoring growth (*n* = 216)**		
Private clinics	152	70.4
Well-baby clinics in PHCs	61	28.2
At home	3	1.4
**Location of monitoring child development (*n* = 179)**		
Private clinics	154	86.0
Well-baby clinics in PHCs	25	14.0
**Reasons for not monitoring children’s growth**^**a**^** (*n* = 178)**		
Long waiting hours	50	28.1
Overcrowding	61	34.3
Busy working	108	60.7
Monitoring is not important	12	6.7

*PHCs* Primary health care centers

^a^More than one answer could be selected

Nearly all (97.3%) of mothers reported their need for a new app to monitor their children’s health. Eighty-eight percent of mothers needed nutrition services whereas, 87.4% reported their need for a developmental milestone’s service. Only 57.9% were satisfied by previous use of child monitoring apps (Table 3).

**Table 3:** Description of caregivers’ demand and needs for the app for children’ health monitoring

	**Frequency (N.)**	**Percent (%)**
**The need for a new app (*n* = 374)**		
In need	364	97.3
Not in need	10	2.7
Services needed in the app* (*n* = 374)		
Nutrition	330	88.2
Developmental milestones	327	87.4
Physical growth	309	82.6
Safety	207	55.3
Immunizations	187	50.0
Psychiatric problems as Autism	2	0.6
Early detection of diseases	1	0.3
Skills development	1	0.3
Behavior modification	1	0.3
**Having smartphones (*n* = 374)**		
Yes	373	99.7
No	1	0.3
**Type of system (*n* = 371)**		
Android	311	83.8
IOS	60	16.2
**Previous use of child health apps (*n* = 374)**		
Yes	123	32.9
No	251	67.1
**Previous use satisfaction (*n* = 147)**		
Satisfied	85	57.9
Not satisfied	23	15.6
Do not know	39	26.5

### Phase 2: application development

**Fig. 2:**
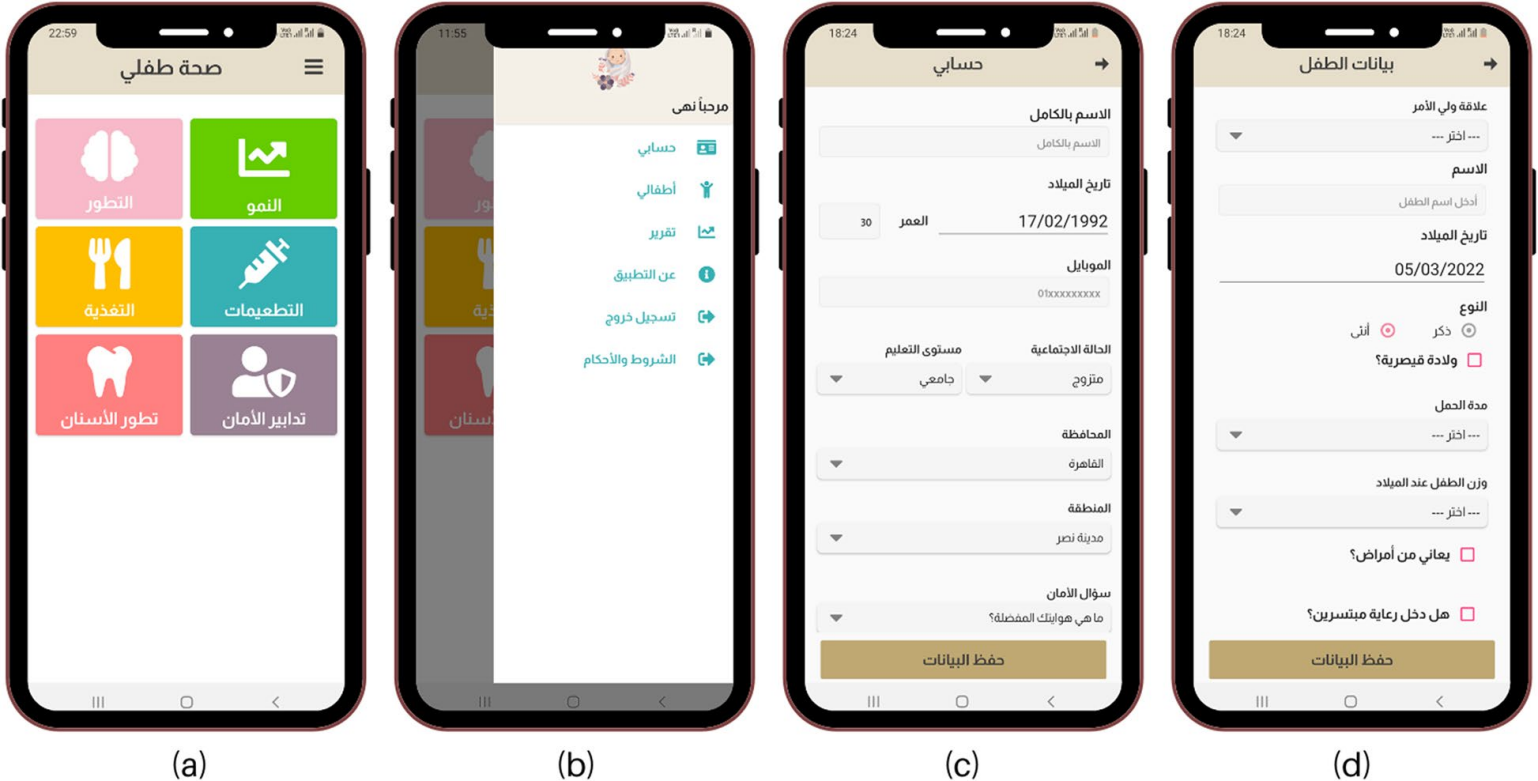
Screenshots of *Sehhat Tefly* app. **a** Main dashboard, **b** Side dashboard, **c** Parent information profile, **d** Child information profile

**Fig. 3:**
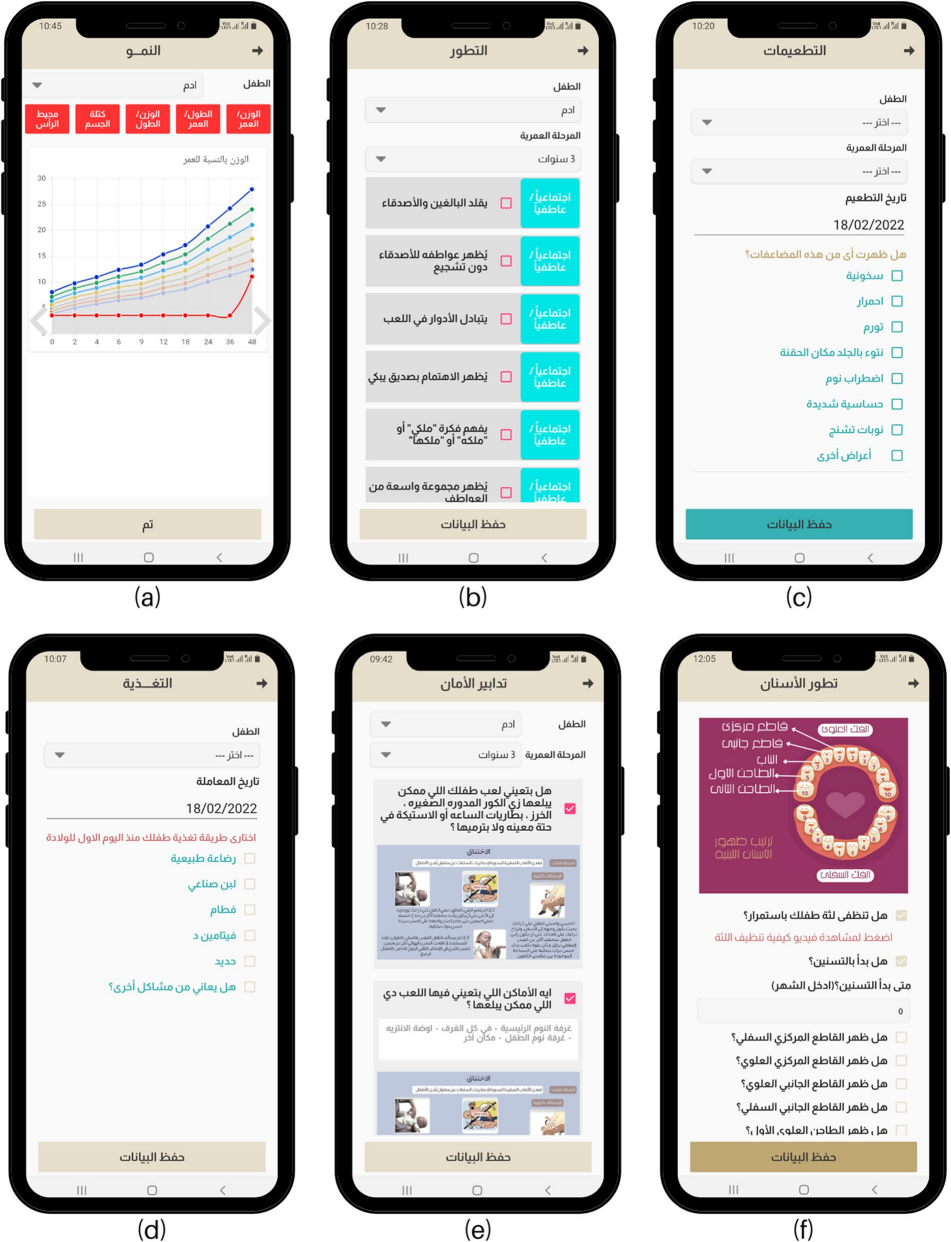
Screenshots of *Sehhat Tefly* app services. **a** Growth charts in physical growth service. **b** Developmental milestones checklist. **c** Immunization service. **d** Nutrition service. **e** Safety service **f** Teething service

The app consists of the following sections:

A. Main dashboard that includes the 6 main services and side dashboard that includes user profile account (parent information), child profile (child information), report, about app, logout button and terms & conditions (Fig. 2). A notification button appears in case of presence of reminders (for scheduled visits or immunization schedule).
B. Parent information: age, marital status, residence (governorate and district), educational level, relation with child and mobile number (Fig. 2).
C. Child information: name, gender, birthday, gestational age, weight at birth (< 1.5 kg – 1.5–2.5 kg – > 2.5), delivery method (normal – caesarian section) and medical conditions (Fig. 2).
D. Well-baby clinic services: Caregivers can monitor growth status, developmental milestones, nutritional status and immunization progress of their child using the seven services of the app (Fig. 3). They can also benefit from the customized feedbacks.
E. App database. The first part “the dynamic app content database” or “back-end system” contained the uploaded data which are a. Residence (27 governorates which were divided further to 295 districts), b. The 10 checklists of CDC’s developmental milestones (a total of 216 milestones), c. Developmental milestones advice according to age (10 groups), d. Nutrition advice according to age (5 groups), e. Categories of growth service according to Z scores (31 categories), f. Analysis questions for growth service according to growth status (60 questions) and g. Advice of growth service according to age and growth status (27 groups). This dynamic feature allows modification of all previously mentioned data without need of app updates as it is automatically updated in user’s mobile phone app. The second part is “users’ information reports” which contains data of users who registered in the app and services they used (Screenshots of the database are illustrated in supplementary file 3).

### Phase 3: beta testing

Beta testing conducted on few app users revealed reporting of some operational malfunctions, overall, participants perceived that the app was of good quality. Difficulties mainly originated from small size health education photos in safety service, not being able to add a new entry and not being able to add child information. These malfunctions were all fixed in the final version of the app by adding zoomable health education photos, replacing the (**+**) button in each service for new entry with the word (Add) and also app automatization where parent information screen led directly to opening of the child information screen for easy accessibility by users.

### Application validation

Ten public health and pediatrics consultants validated the app using MARS. The mean scores ratings for engagement, functionality, aesthetics and information were 3.5, 3.7, 3.9 and 3.7 respectively. The app quality mean was rated 3.7/5 while the subjective quality score mean was 3.2/5 (Table 4).

**Table 4:** Application rating via MARS questionnaire by public health and pediatrics experts (score out of 5)

**#**	Consultant specialty	Engagement score	Functionality score	Aesthetics score	Information score	App quality mean	Subjective quality mean
**1**	**Public health**	4.4	2.8	3.7	4.4	3.8	3.3
**2**	**Public health**	3.0	3.0	4.0	2.8	3.2	2.0
**3**	**Pediatrics**	2.2	3.8	3.7	3.7	3.3	2.0
**4**	**Public health**	3.8	4.5	4.3	3.7	4.1	3.0
**5**	**Pediatrics**	3.6	4.3	3.7	3.2	3.7	3.3
**6**	**Public health**	3.6	3.8	3.3	3.5	3.5	4.0
**7**	**Pediatrics**	3.6	4.0	4.7	4.2	4.1	4.0
**8**	**Pediatrics**	3.8	3.8	4.0	3.5	3.8	3.5
**9**	**Public health**	3.8	3.5	3.3	4.0	3.7	4.0
**10**	**Public health**	3.6	3.8	4.7	4.3	4.1	2.8
	Mean ± SD	3.5 ± 0.58	3.7 ± 0.52	3.9 ± 0.50	3.7 ± 0.51	3.7 ± 0.32	3.2 ± 0.75

### Phase 4: application implementation and usage

**Fig. 4:**
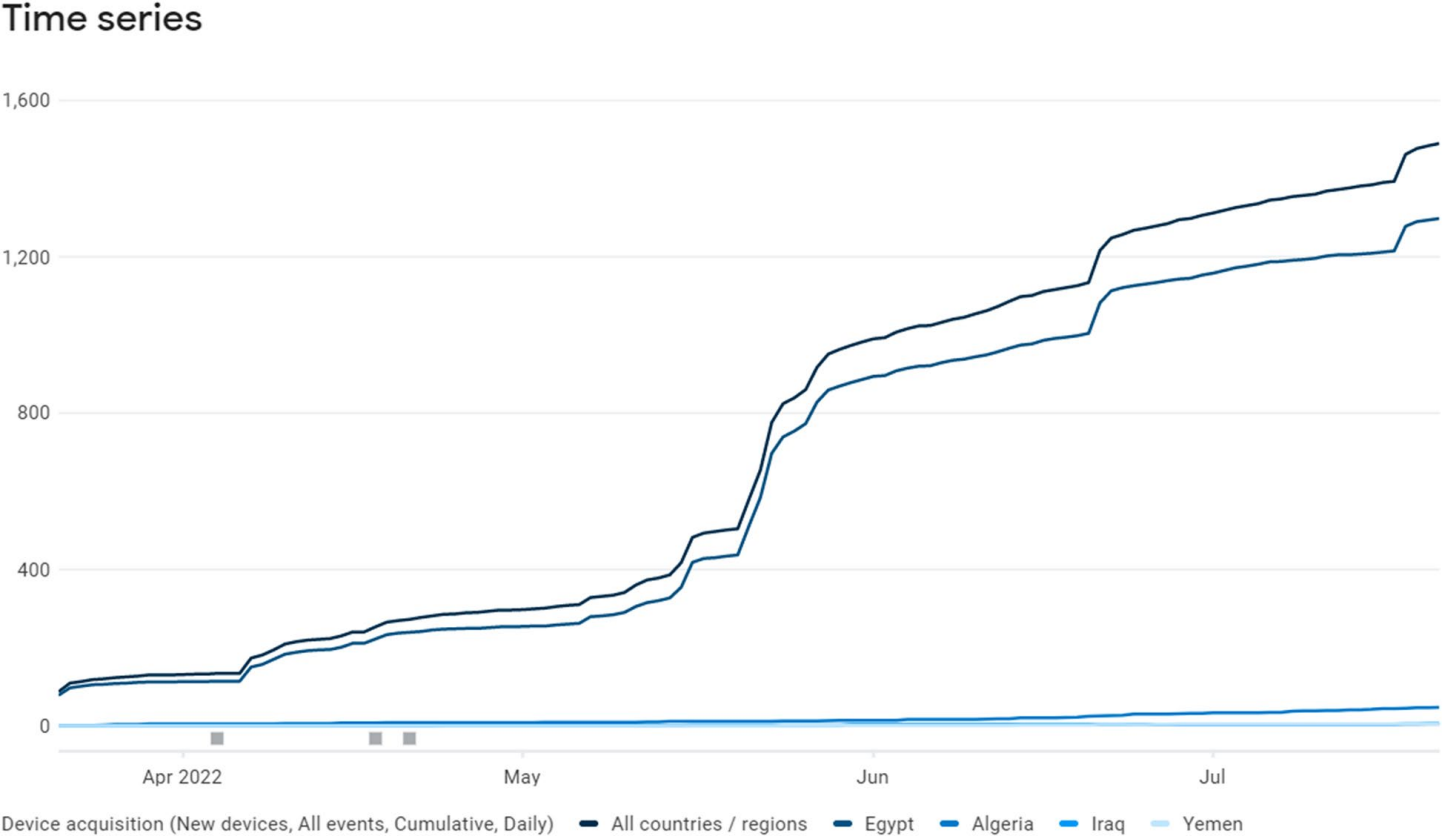
Cumulative user acquisition of the app (Screenshot from Google Play Console) [17]

The app was launched and made available to the public on Android platform. Four-month period was needed to reach a quota sample of 500 children (100 in each age category. In this period, the app had been downloaded 1445 times, 943 of them registered in the app with an overall response rate of 65.3%. In this period, store listing conversion rate (store listing acquisition divided by store listing visitors) was 59.1%. Figure 4 shows the increase in the downloads throughout this period.

## Discussion

To our knowledge, this is the first Egyptian study that describes development, implementation and validation of a well-baby mobile app. We started our needs assessment by undergoing app research in Google Play store, we found that not any Arabic app provided a comprehensive approach to well-baby clinic services. All (100%, 13/13) were in the form of written articles that offered no interactive features for parents. None of them (0%, 0/13) were developed by a recognized health organization. Almost all of them (92.3%, 12/13) included ads for financial gains. Participants in the “Online assessment survey” gave us insights into the requirements of the app that suited them. Most of them (97.3%) were in need of a new app to regularly follow their children’s health and also reported the need for nutrition, developmental milestones and physical growth services.

While traditional well-baby clinics provide outpatient health care and primary preventive activities for children, they have numerous flaws. They are not comprehensive as they primarily focus on immunization services neglecting other critical aspects of well-baby assessment such as physical growth, for example, only 69% of children’s growth were plotted on charts in an Alexandrian PHC center [18]. Traditional services also lack tracking babies’ dental health [19] and developmental milestone tracking [3]. As for feasibility, the paper-based follow up cards impose significant publishing and distribution costs. Parents must remember to bring the card to well-child visits for the best care. Also, it can be lost because of personal problems or environmental disasters. Dissatisfaction from well-baby clinics stems primarily from the long waiting hours, high travel costs and unsanitary environment [18, 20]. To this end, mHealth technologies can be used to overcome the disadvantages of the traditional services. Further, it is an affordable way of transforming healthcare delivery services as it provides supervised healthcare and continuous monitoring specially in the Arab countries [21].

### App development

Taking into consideration caregiver’s requirements and app research, we designed the first draft of the app after few brainstorming discussions. In a 4 months period, after several modifications and tailoring, we reached the final design of the app *Sehhat Tefly*, and it was ready to be developed. The app offers comprehensive services of child’s nutrition, development, safety, teething, growth and immunizations for parent supervision with no financial burden. Elements of this app are available in other child monitoring apps; however, each have three or less services at a time and include subscriptions averaged 57 dollars every year, ranging from 3 to 225 dollars every year, according to a review on English apps [22].

*Sehhat Tefly* app was developed by academic health care providers in the field of public health and computer science app developers. The app provides evidence-based content and respects privacy of users’ data. Privacy and protection of data were ensured by applying authentication. Thus, only the research team were granted access to this data. App users had their own usernames and passwords to access their profile in the app. The app’s data were not shared with other parties or service settings. An Australian study reported that only 40% of the apps involved health care professionals and provided evidence-based content, and only 30% implemented user privacy security measures [23]. Health care providers should be equipped with strategies to guide parents safe use of technology and to support parents in their search for evidence based apps. They can encourage parents to look for the privacy policy of apps to ensure that their personal information is not shared with third parties and to verify the quality of health information [24]. Since caregivers generally rely on the content present in the app store to make informed decisions about which apps to download according to their needs [25], we included all the app details in the Google play store download page of the app mentioning all referencing agencies as WHO and CDC.

The app offers tailored feedbacks and interactive features that have more persuasion than standalone apps (with no interactivity) [26]. Non-feedback apps constituted about 63.7% of the apps included in review by DeWitt et al. [22]. The repot section of the app also gathers all health data of the child in one place, this single place storage would make it easy for parents to share their children’s health data with their health care providers.

Well babies’ health services have been introduced in previous apps. Some assessed child’s risk of obesity as “Memphis FitKids” app that used collected information to create a report with tailored recommendations on how to make healthy changes in weight and lifestyle of children [27]. Two other studies have shown that apps can help improve caregiver’s understanding of growth charts [28] and detection of abnormal child growth early in life [29]. Other studies addressed dental caries and oral health, the apps improved the oral health knowledge and practice of caregivers while oral hygiene as a result of plaque control was superior in children of mothers using apps containing games [30, 31].

Another study included developmental screening tool, but unlike our app, text messages were used. The app was considered usable and acceptable in low-income setting [32]. Other mhealth tool sent reminders of immunization schedule to parents in the form of SMS and reported that it was a feasible tool for increasing routine immunization coverage in Pakistan [33].

### App validation

Since app quality is one of the most influencing factors in the success of mHealth apps, validation of this app was conducted by public health and pediatrics experts in the field of child health using MARS questionnaire [16]. The app quality mean was rated 3.7/5 while the subjective quality score was 3.2/5. The consultants thought the app was comprehensive, relevant and useful to parents for keeping track of their baby’s health in Arabic language using explanatory videos and photos. They recommended adding some features as gamified health education messages, adding the additional recommended vaccines next to the compulsory vaccination schedule and adding illustrative photos to each of the developmental milestones and vaccine side effects.

Mobile apps can support parents to identify and document patterns in their child’s health that would otherwise go undetected and encourage caregivers to seek medical attention. At the same time, these apps can risk pathologizing health behaviors, raising unfounded fears, performing self-management, and adding stress to families [22]. There had been numerous tracking apps for children’s health as “Child Growth Tracker” app (rated 4.5/5 by MARS) which was simple and easy to use because it offers multiple evidence-based scales for tracking a child's growth, but it was solely designed for recording infants’ growth such as height, weight, head circumference, and BMI ignoring other important options as information, immunization, developmental milestones or reminders [24].

Other successful story in the field of child health apps is “KhunLook” app which included seven key features: growth and nutrition, development, immunizations, oral health, reminders for the next appointment, memories and health advice [34]. Other apps showed success in monitoring vaccination schedule, nutritional health, developmental milestones and injury prevention of children [8, 35–37].

*Sehhat Tefly* app is categorized as being an “academic app”. In contrast, “MyMedela Breastfeeding Companion” app (rated 4.3/5 by MARS), a commercial app, was designed for breastfeeding mothers to promote Medela products; however, users are not required to buy any Medela products in order to use the app. It provides basic activity tracking and some information but offers no immunization service or scheduled child visits. Caution is advised while using the app given that the information provided might be influenced by financial gains [24]. Commercial apps, which are more popular among parents than academic ones [38], may jeopardize privacy and security of users’ information by leaking or selling personal data to third parties. Commercial parenting apps may not be developed and validated with the same rigor as apps developed based on research [22]. Unfortunately, Google Play store and Apple store do not have extensive guidelines or regulatory supervision over parenting apps aside from legal restrictions on claims of specific health outcomes [39].

### App database

The primary objective of the app is for child health supervision by caregivers, but it can also be used as a database for epidemiologic surveillance. The app’s developed database can measure important growth indicators as stunting, overweight, obesity & underweight [7] and nutritional indicators as ever breastfeeding, exclusive breastfeeding first 6 months and weaning. Digital data collection has been shown to have fewer errors when compared to manual data reporting [40] and considered as a faster tool of surveillance of nutritional problems [41]. Previous app called “Information for Action” succeeded in repackaging routinely collected child growth and development assessment data into useful decision support for caregivers and program planners [42].

### App usage

*Sehhat Tefly* app was launched on Google play and was downloaded 1445 times in a 4-months period. At the end of this period, the number of active users were 667 (user loss ratio = 53.8%). This is attributed to the fact that there may be some difficulties in app use. Therefore, work must be done to improve ease of use in order to fully benefit from the important functions provided by the app for proper child health follow up. Collecting feedback from future users will help in updating the app, modifying existing features and adding new features, for example, in-app tutorials, incorporating text messages, adding allergy & medication sections.

### Strengths

Strengths of this paper include a full description of *Sehhat Tefly* app development process, which could be replicated for future health system development in other LMICs.

### Limitations

The search for apps was restricted to Google play store not including other engines as the Apple store. For user requirement, a quantitative research was done, a qualitative research would have added new insights into the app development. While *Sehhat Tefly* app has been beta-tested, resulting in improvements, extensive evaluation was not illustrated as part of this manuscript. Reference means and standard deviations extracted were selected according to well-baby visits schedule, so in between those visits, the age of child had to be approximated to the nearest available reference for calculation of the Z scores which might have limited the accuracy of the calculations. Further, the app only offered in-app notification/ reminder as push notifications and text messages were too expensive to incorporate. Finally, the app was launched only on Google Play Store due to scarcity of resources.

### Further directions

The potential of this mHealth app has not been fully established yet. Further studies should be implemented to assess accessibility and usability of the app to explore difficulties and barriers faced by users in different services using of focus group discussions (FGDs). Comparative studies should be conducted involving a large number of users to assess its effect on behavioral change and health outcomes. Further, validation studies should be designed to evaluate the congruence of the assessment between the app and the traditional medical services.

## Conclusions

*Sehhat Tefly* app can meet the need for a free, easy and accessible tool for caregivers for monitoring their children’s wellbeing. Collection of more data over the next years will provide more insights for integrating mHealth solutions with well-baby clinics and for making caregivers involved in monitoring their children.

## Supplementary Information

**Additional file 1.** App user flowchart diagram.

**Additional file 2.** Description of Arabic apps concerning well-babies’ health and wellbeing.

**Additional file 3.** Dynamic app content database and users’ information reports database (Screenshots from the app’s database).

## Data Availability

Project name: Development of well-baby clinic mobile system “*Sehhat Tefly*” Project home page: https://play.google.com/store/apps/details?id=com.itspace.mybaby. Operating system(s): Android Programming language: Java License: freely available Any restrictions to use by non-academics: not restrictions

## Abbreviations

AAB: Android App Bundle
ADA: American dental association
ADDIE: Analysis Design Development Implementation Evaluation
API: Application programming interfaces
App: Application
BMI: Body mass index
CDC: Centers of disease control and prevention
ID: Identification number
IT: Information technology
LMICs: Low middle income countries
MARS: Mobile application rating scale
mHealth: Mobile health
PHCs: Primary health care centers
SD: Standard Deviation
WHO: World Health Organization
