# Supplementary figures and images for: A novel wide scale well-baby clinic mobile application: an Egyptian pilot study
Source: BMC Health Serv Res. 2023 Jun 24;23:687. doi: 10.1186/s12913-023-09720-0 (PMC10290293; doi:10.1186/s12913-023-09720-0)

**Supplementary file (1):** App user flowchart diagram


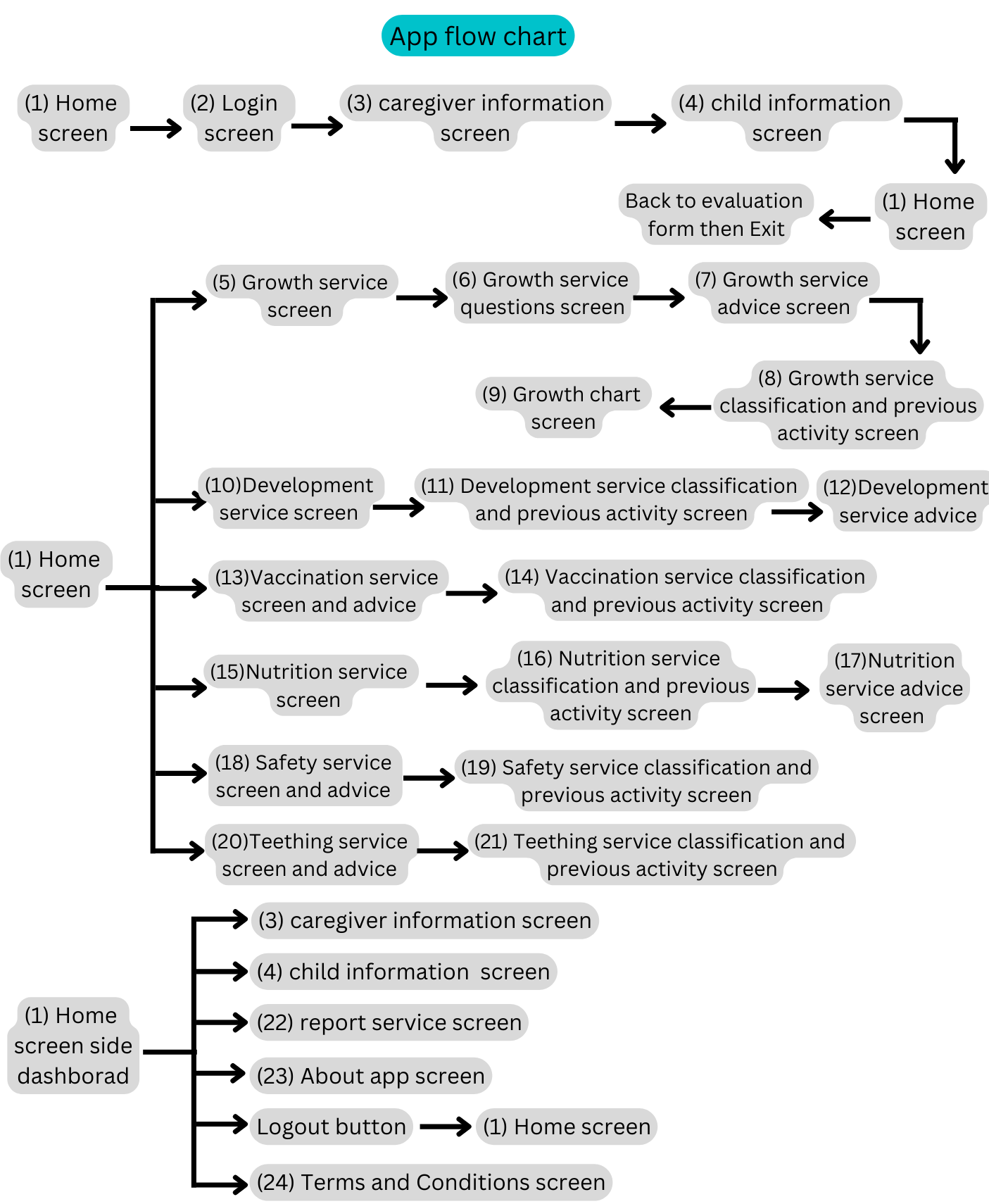

Supplement: Supplementary file 1 — Additional file 1. App user flowchart diagram. [file 12913_2023_9720_MOESM1_ESM.docx]
