# Supplementary material for: A novel wide scale well-baby clinic mobile application: an Egyptian pilot study
Source: BMC Health Serv Res. 2023 Jun 24;23:687. doi: 10.1186/s12913-023-09720-0 (PMC10290293; doi:10.1186/s12913-023-09720-0)
Supplement: Supplementary file 2 — Additional file 2. Description of Arabic apps concerning well-babies’ health and wellbeing. [file 12913_2023_9720_MOESM2_ESM.docx]

**Supplementary file 2**

| Description of Arabic apps concerning well-babies’ health and wellbeing | | | | | |
| --- | --- | --- | --- | --- | --- |
| **Application** | **Brief description** | **Disadvantages** | **Downloads** | **Release** | **Rating**† |
| ماى بيبى  “My baby” | - - - - Nutritional advice       - Breastfeeding advice       - Parenting advice       - Frequent diseases       - Developmental stages       - Vaccination table       - Size ♂ 5.2 MB | - - - - Contains ads       - No references are found (not scientific based)       - Narrative (no interactive features)       - Static vaccination table with no side effects treatment       - No teething or safety service | 100,000 + | 2017  Last updated*:  2017 | 4.7 |
| تربية الرضيع بدون انترنت  “Infant raising without internet” | - - - - Nutritional advice       - Breastfeeding       - Weaning       - Sleep meter       - Size 5.2 MB | - - - - Contains ads       - No references are found (not scientific based)       - Narrative (no interactive features)       - No growth or developmental service | 100,000 + | 2016  Last updated:  2017 | 4.1 |
| انت و طفلك  “You and your baby” | - - - - Feeding tracker       - Sleep       - Development of child       - Vaccination table       - Sleep meter       - Weight and height tracking       - Size 28 MB | - - - - Contains ads       - No references are found (not scientific based)       - Narrative developmental milestones (no interactive features)       - Static vaccination table with no side effects treatment       - Weight and height tracking with no interpretation of growth status       - No safety or teething service | 100,000 + | 2014  Last updated:  2019 | 4.1 |
| دليلك الشامل لتغذية الطفل  “Your comprehensive guide to baby feeding” | - - - - Nutritional advice       - Size 23 MB | - - - - Contains ads       - No references are found (not scientific based)       - Narrative (no interactive features)       - Not comprehensive (only nutrition) | 5,000 + | 2021  Last updated:  2021 | - |
| جدول تغذية الطفل 6 شهور  “Baby feeding schedule 6 months” | - - - - Nutritional advice       - Size 16 MB | - - - - Contains ads       - No references are found (not scientific based)       - Narrative (no interactive features)       - Not comprehensive (only nutrition) | 1,000 + | 2022  Last updated:  2022 | - |
| طفلك خطوة بخطوة  “Your child step by step” | - - - - Nutritional advice       - Breastfeeding       - Frequent diseases       - Vaccination table       - Growth scales by month       - Teething       - Vaccination table       - Size 7.1 MB | - - - - Contains ads       - No references are found (not scientific based)       - Narrative (no interactive features)       - Static vaccination table with no side effects treatment       - Only normal ranges of weight and height are present with no z score calculation or interpretation of growth status       - No safety service | 100,00 + | 2017  Last updated:  2019 | 4.4 |
| دليل الطفل  “Baby guide” | - - - - Nutritional advice       - Teething       - Development (hearing- speaking- vision- crawling- walking- sitting)       - Size 3 MB | - - - - Contains ads       - No references are found (not scientific based)       - Narrative (no interactive features)       - Narrative developmental milestones (no interactive features)       - No growth or safety service | 100,000 + | 2013  Last updated:  2019 | 4.1 |
| وجبات الرضع – سهلة و صحية  “Infant meals - easy and healthy” | - - - - Nutritional recipes       - Size 2.6 MB | - - - - Contains ads       - No services       - Not comprehensive | 10,000 + | 2018  Last updated:  2018 | 3.7 |
| أنا و طفلى  “Me and my baby” | - - - - Development according to age       - Activities according to age       - Size 4.6 MB | - - - - Contains ads       - No references are found (not scientific based)       - Narrative developmental milestones (no interactive features)       - Not comprehensive | 500,000 + | 2013  Last updated:  2022 | 4.2 |
| نصايح للأم و الرضيع حديث الولادة  “Advice for mothers and newborn infant” | - - - - Nutritional advice       - Size 16 MB | - - - - Presence of ads       - No references are found (not scientific based)       - No services       - Not comprehensive | 5,000 + | 2021  Last updated:  2022 | 4.2 |
| وجبات الرضع  “Infant meals” | - - - - Nutritional recipes       - Size 4.1 MB | - - - - Contains ads       - No services       - Not comprehensive | 100,000 + | 2016  Last updated:  2018 | 4.3 |
| طفلى شهر بشهر  “My baby- month by month” | - - - - Development of child by month       - Size 18 MB | - - - - Contains ads       - No references are found (not scientific based)       - Narrative (no interactive features)       - Narrative developmental milestones (no interactive features)       - No growth or immunization or teething or nutritional service | 1,000 + | 2022  Last updated:  2022 | - |
| تطبيق صحة الطفل  Child Care  “Child health app  Child Care” | - - - - Narrative articles on nutrition and food pyramid       - Vaccination reminder       - General articles on psychological health       - Activities according to age       - Size 15 MB | - - - - No references are found (not scientific based)       - No services       - Not comprehensive | 1,000 + | 2019  Last updated:  2019 | - |
| *Last updated: The better the recent.  †The average rating score of the app on a scale ranging from 1 (very dissatisfied) to 5 (very satisfied), the higher the better, new apps are not rated.  ♂ Size in Megabytes, the smaller the better. | | | | | |
