# Supplementary material for: A novel wide scale well-baby clinic mobile application: an Egyptian pilot study
Source: BMC Health Serv Res. 2023 Jun 24;23:687. doi: 10.1186/s12913-023-09720-0 (PMC10290293; doi:10.1186/s12913-023-09720-0)

**Supplementary file (3):** Dynamic app content database and users’ information reports database (Screenshots from the app’s database)


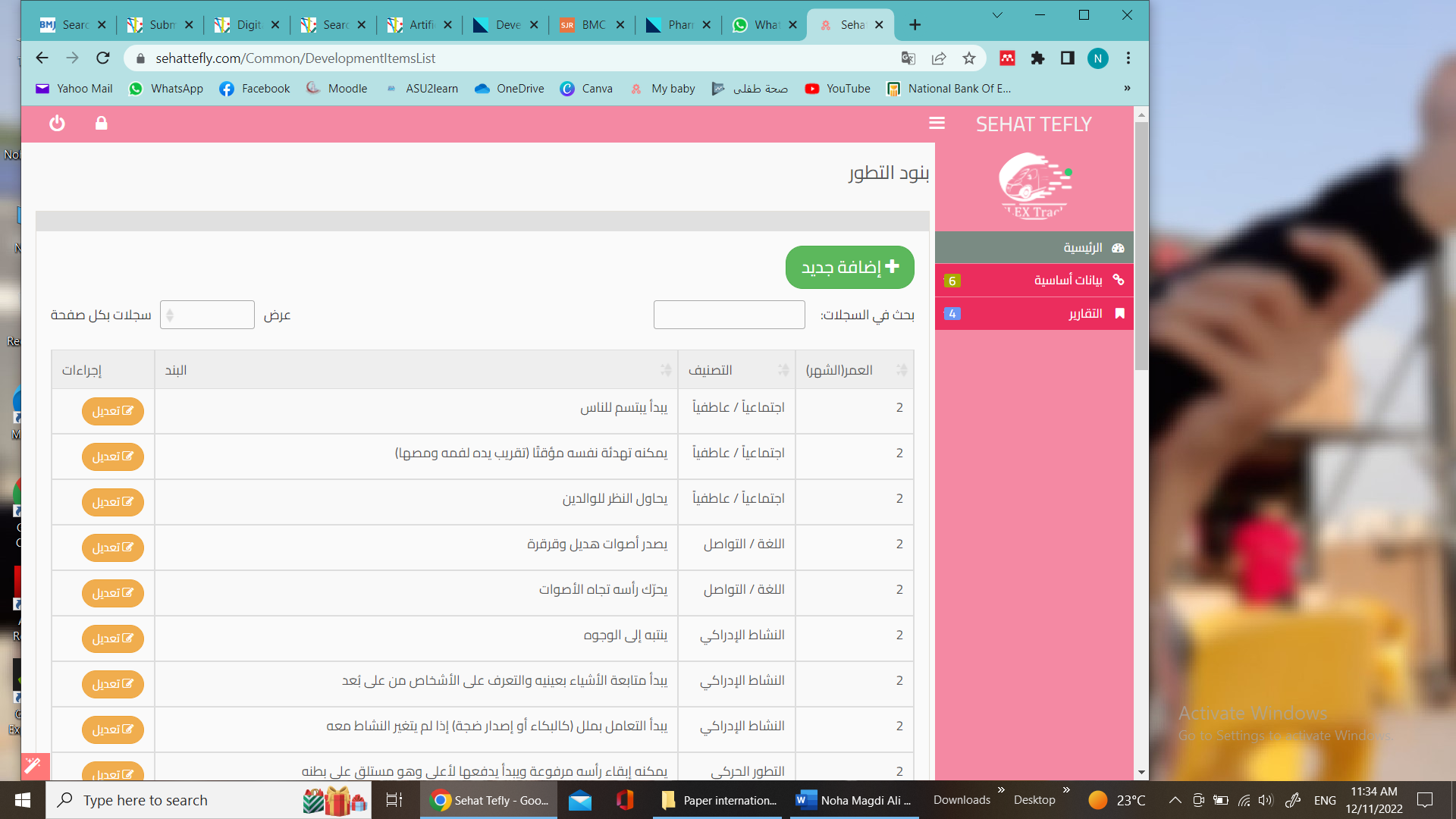


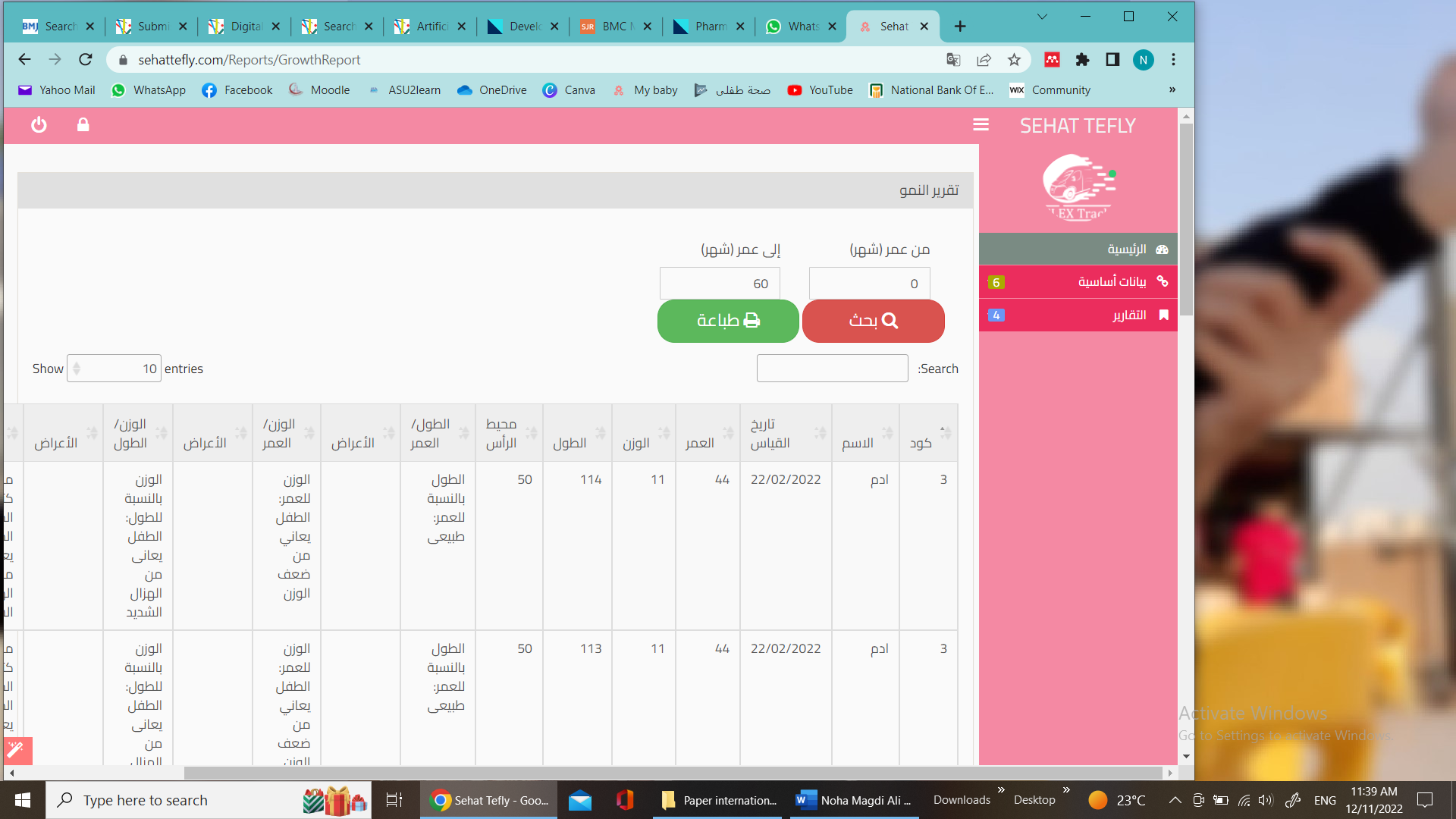

Supplement: Supplementary file 3 — Additional file 3. Dynamic app content database and users’ information reports database (Screenshots from the app’s database). [file 12913_2023_9720_MOESM3_ESM.docx]
